# A model to predict disease progression in patients with autosomal dominant polycystic kidney disease (ADPKD): the ADPKD Outcomes Model

**DOI:** 10.1186/s12882-017-0804-2

**Published:** 2018-02-13

**Authors:** Phil McEwan, Hayley Bennett Wilton, Albert C. M. Ong, Bjarne Ørskov, Richard Sandford, Francesco Scolari, Maria-Cristina V. Cabrera, Gerd Walz, Karl O’Reilly, Paul Robinson

**Affiliations:** 1 Swansea Centre for Health Economics, Swansea University, Swansea, UK; 2 Health Economics and Outcomes Research Ltd, Cardiff, UK; 3 Academic Nephrology Unit, University of Sheffield Medical School, Sheffield, UK; 4 Sheffield Kidney Institute, Sheffield Teaching Hospitals NHS Foundation Trust, Sheffield, UK; 5 Department of Internal Medicine, Section of Nephrology, Zealand University Hospital, Roskilde, Denmark; 6 Academic Laboratory of Medical Genetics, Addenbrooke’s Treatment Centre, Cambridge, UK; 7 Department of Nephrology, University of Brescia, Brescia, Italy; 8 Hospital Universitario La Paz, Madrid, Spain; 9 Department of Nephrology, University Medical Centre Freiburg, Freiburg, Germany; 10 Otsuka Pharmaceutical Europe Ltd, Gallions Wexham Springs, Framewood Road, Wexham, SL3 6PJ UK

**Keywords:** Disease modelling, End-stage renal disease, ESRD, Kidney volume, Renal progression, Renal function decline

## Abstract

**Background:**

Autosomal dominant polycystic kidney disease (ADPKD) is the leading inheritable cause of end-stage renal disease (ESRD); however, the natural course of disease progression is heterogeneous between patients. This study aimed to develop a natural history model of ADPKD that predicted progression rates and long-term outcomes in patients with differing baseline characteristics.

**Methods:**

The ADPKD Outcomes Model (ADPKD-OM) was developed using available patient-level data from the placebo arm of the Tolvaptan Efficacy and Safety in Management of ADPKD and its Outcomes Study (TEMPO 3:4; ClinicalTrials.gov identifier NCT00428948). Multivariable regression equations estimating annual rates of ADPKD progression, in terms of total kidney volume (TKV) and estimated glomerular filtration rate, formed the basis of the lifetime patient-level simulation model. Outputs of the ADPKD-OM were compared against external data sources to validate model accuracy and generalisability to other ADPKD patient populations, then used to predict long-term outcomes in a cohort matched to the overall TEMPO 3:4 study population.

**Results:**

A cohort with baseline patient characteristics consistent with TEMPO 3:4 was predicted to reach ESRD at a mean age of 52 years. Most patients (85%) were predicted to reach ESRD by the age of 65 years, with many progressing to ESRD earlier in life (18, 36 and 56% by the age of 45, 50 and 55 years, respectively). Consistent with previous research and clinical opinion, analyses supported the selection of baseline TKV as a prognostic factor for ADPKD progression, and demonstrated its value as a strong predictor of future ESRD risk. Validation exercises and illustrative analyses confirmed the ability of the ADPKD-OM to accurately predict disease progression towards ESRD across a range of clinically-relevant patient profiles.

**Conclusions:**

The ADPKD-OM represents a robust tool to predict natural disease progression and long-term outcomes in ADPKD patients, based on readily available and/or measurable clinical characteristics. In conjunction with clinical judgement, it has the potential to support decision-making in research and clinical practice.

**Electronic supplementary material:**

The online version of this article (10.1186/s12882-017-0804-2) contains supplementary material, which is available to authorized users.

## Background

Autosomal dominant polycystic kidney disease (ADPKD) is the most common monogenic kidney disease, and the leading inheritable cause of end-stage renal disease (ESRD) among adults [1, 2]. The disease arises from genetic mutations in *PKD1* (85% of cases) and *PKD2* (15% of cases), which cause progressive bilateral renal cyst formation, kidney enlargement, fibrosis, chronic kidney disease (CKD) and renal failure. While ADPKD may present in utero and during childhood, early-stage disease is often asymptomatic and undiagnosed due to compensatory glomerular hyperfiltration [1, 3]. In later stages of ADPKD, the irreversible loss of functional glomeruli exhausts compensatory mechanisms, leading to a detectable decline in glomerular filtration rate (GFR) during the third and fourth decades of life [1, 3, 4]. The natural course of ADPKD towards renal failure is heterogeneous between patients and variable over time; however, the average age at which patients commence renal replacement therapy (RRT) for progressive disease falls between 55 and 60 years across European countries [5, 6].

The social and economic burden of ADPKD on patients and healthcare systems is largely driven by the incurable deterioration of kidney function, and the provision of RRT to patients who ultimately progress to ESRD [1, 2, 7]. Early identification of ADPKD patients with rapidly progressing disease may facilitate the selection of those most likely to benefit from treatment in clinical trials and clinical practice; thus, improving the cost-effectiveness and benefit-to-risk ratio of novel therapies in a population with high unmet need [1, 8, 9]. A systematic literature review by Woon et al. identified age at diagnosis and total kidney volume (TKV) as the most commonly cited prognostic indicators associated with rapid ADPKD progression [10]; additional factors reported in the literature include baseline GFR, male gender and *PKD1* mutation [1, 3, 10].

Until recently, treatment strategies for ADPKD were limited to managing its clinical manifestations of hypertension, pain, urinary tract infection, and kidney stones. In 2015, tolvaptan (a selective vasopressin V2 receptor antagonist) received marketing authorisation from the European Medicines Agency (EMA), to delay ADPKD progression in adults with CKD stage 1–3 and evidence of rapidly progressing disease [11]. Recommendations by the European Renal Association-European Dialysis and Transplant Association (ERA-EDTA) include a hierarchical treatment algorithm to identify those rapidly progressing patients most likely to benefit from tolvaptan, based on estimated GFR (eGFR) decline, documented TKV growth and other clinical factors [8].

Clinical evidence that informed EMA and ERA-EDTA guidance was derived from the phase 3, double-blind, placebo-controlled Tolvaptan Efficacy and Safety in Management of Autosomal Dominant Polycystic Kidney Disease and Its Outcomes trial (TEMPO 3:4; ClinicalTrials.gov identifier NCT00428948) [12]. The clinical effectiveness of tolvaptan was evaluated using study endpoints of TKV (measured using a gold-standard magnetic resonance imaging protocol) and eGFR, which are established determinants of ADPKD progression and kidney function, respectively [8, 13]. Data was collected from 1445 patients across 129 international sites over 3 years; thus, TEMPO 3:4 represents one of the largest sources of high-quality patient-level data currently available in the field of ADPKD.

The development of therapies that delay ADPKD progression drive the requirement for resources that identify patients eligible for treatment and optimise clinical decision-making. Since early-stage ADPKD is largely undiagnosed, and disease progression towards ESRD is heterogeneous and prolonged over several decades, the size and length of clinical trials in ADPKD are insufficient to capture the natural history of the disease [10]. Alternatively, simulation modelling can predict disease progression over time horizons longer than that feasible in clinical trials, and may therefore represent a useful tool to model the natural rate of renal decline, particularly when measurable and/or observable patient characteristics are used as inputs. Using patient-level data from the placebo arm of TEMPO 3:4, the aim of the present study was to develop and validate a natural history model that simulated disease progression according to this principle, and predicted long-term outcomes across a range of ADPKD patient profiles.

## Methods

### ADPKD progression equations

To identify relationships between TKV, eGFR and available patient risk factors, exploratory analysis of patient-level data from the TEMPO 3:4 placebo arm (Table 1) was conducted using R version 2.12.2. Candidate prognostic variables assessed within a multivariable regression framework included those identified in the literature [10], agreed upon by clinical experts in ADPKD management, and available within the TEMPO 3:4 dataset: baseline age, gender, child bearing age, ethnicity, region, country, TKV (analysed at baseline, 12, 24 and 36 months) and eGFR (analysed at corresponding timepoints). Candidate variables were entered into the model simultaneously, and the step AIC approach [14] was used to identify candidate models and test for interactions between covariates.

**Table 1:** Baseline patient characteristics (post-randomisation) and changes in TKV and eGFR, as observed in the placebo arm of TEMPO 3:4 study [12, 39]

	Placebo arm (*N* = 484)	
Male gender (n, %)	251 (51.9)	
TKV (mL)		
Baseline (mean, SD)	1667.5 (873.1)	
Annual change (mean, SD)	114.4 (113.2)	
eGFR^a^	1/SC ([mg/mL]^−1^)	CKD-Epi (mL/min/1.73 m^2^)
Baseline (mean, SD)	104.30 (33.87)	82.14 (22.73)
Annual change (mean, SD)	−3.682 (6.361)	−3.568 (4.495)

*1/SC* reciprocal of serum creatinine, *CKD-Epi* Chronic Kidney Disease Epidemiology Collaboration, *eGFR* estimated glomerular filtration rate, *SD* standard deviation, *TKV* total kidney volume

^a^eGFR was measured using both the reciprocal of serum creatinine (1/SC) and Chronic Kidney Disease Epidemiology Collaboration (CKD-Epi) equation [12]

Selected covariates were incorporated within a two-step statistical model that predicted annual changes in TKV (Eq. 1) and eGFR (Eq. 2), based on statistical significance and clinical relevance. A natural log transformation was applied, where it provided a better fit and improved regression diagnostics. Subsequently, multivariable regression equations were fitted to mean annual changes in TKV + 500 and eGFR + 60, to avoid taking the natural log of a negative number.

Renal function in TEMPO 3:4 was assessed using two measures of eGFR [12]. The secondary study endpoint was the change in kidney function according to the reciprocal of serum creatinine, while eGFR based on the Chronic Kidney Disease Epidemiology Collaboration (CKD-Epi) equation [15] was used to ascertain CKD stage at baseline, in line with clinical guidelines [13]. In the present study, changes in eGFR were modelled based on the reciprocal of serum creatinine data from TEMPO 3:4. However, the impact of using CKD-Epi equation estimates of GFR to fit the coefficients of the eGFR progression equation was additionally assessed in sensitivity analysis (Additional file 1).

**Eq.** 1 Annual change in TKV equation implemented within the ADPKD-OM1

*TKV, total kidney volume; ΔTKV, 1-year change in TKV; α, age coefficient; β, TKV coefficient; γ, female coefficient; δ, age:LnTKV coefficient; λ, intercept.*

**Eq.** 2 Annual change in eGFR equation implemented within the ADPKD-OM2

*eGFR, estimated glomerular filtration rate; ΔeGFR, 1-year change in eGFR; TKV, total kidney volume; β, Ln(TKV) coefficient; λ, intercept.*

### ADPKD Outcomes Model

**Fig. 1:**
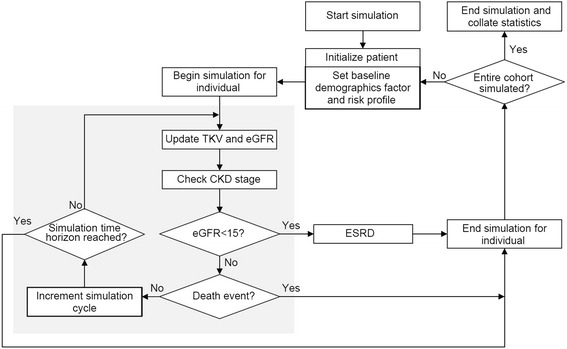
Flow diagram of patient simulation through the ADPKD Outcomes Model. CKD: chronic kidney disease; eGFR: estimated glomerular filtration rate; ESRD: end-stage renal disease; TKV: total kidney volume

Disease progression equations formed the basis of the ADPKD Outcomes Model (ADPKD-OM); a fixed-time increment stochastic simulation model, implemented in Microsoft Excel and coded in Visual Basic for Applications. The ADPKD-OM conducted patient-level simulations to predict the natural history of ADPKD, and aggregated estimates across a hypothetical cohort of up to 10,000 patients. For each year of the simulated time horizon, disease progression equations predicted the natural course of ADPKD progression and produced non-linear trajectories of TKV and eGFR. Simulated patients progressed through the model over a maximum horizon of 80 years (i.e. lifetime); from baseline, between CKD stages, and until the onset of ESRD (eGFR <15 mL/min/1.73 m^2^) or death, whichever occurred first (Fig. 1). All-cause mortality was modelled according to gender-specific life tables obtained from the UK Office for National Statistics [16]. Key outputs of the ADPKD-OM, reported across the simulated cohort, included the incidence of ESRD (over a lifetime horizon or by a pre-defined age), average age at ESRD onset, and distribution of time spent across CKD stages.

Uncertainty in model predictions of ADPKD progression was accounted for; thus, patients with the same baseline characteristics could incur different TKV and eGFR trajectories, reflective of natural inter-patient variability. This was achieved by sampling the ADPKD progression equation coefficients from multivariable normal distributions, using variance covariance matrices associated with each regression equation (Additional file 1: Tables S1 and S2).

### Model validation

External validation exercises tested the accuracy of the ADPKD-OM to predict natural disease progression, and investigate the generalisability of its predictions to alternative ADPKD patient populations. In the absence of a lifetime study of ADPKD patients, model predictions were validated against alternative study cohorts that differed with respect to patient characteristics, disease stage and rate of progression. Briefly, ADPKD-OM progression equations were first compared against those derived from annual TKV and eGFR changes observed in the Consortium for Radiologic Imaging Studies of Polycystic Kidney Disease (CRISP I) study, a population not fully enriched for patients with rapidly progressing ADPKD [17, 18]. Modelled trajectories of TKV and eGFR were then compared against the results of the Halt Progression of Polycystic Kidney Disease (HALT-PKD) studies, to validate ADPKD-OM predictions in patients with early-stage (Study A) and late-stage ADPKD (Study B) [19, 20]. To further validate the ADPKD-OM in patients with late-stage disease in clinical practice, simulated eGFR trajectories were compared against observed data for 64 patients selected from The Health Improvement Network (THIN) database, over 6 years prior to ESRD [21]. Further details of the model validation exercises performed in this study are described in Additional file 1.

### Model application

Following validation, the ADPKD-OM was used to simulate disease progression in a hypothetical cohort with baseline characteristics matched to the overall TEMPO 3:4 study population (Table 2). The underlying variability of disease progression was assessed by simulating individual patients drawn from a cohort with the described baseline characteristics, to report standard deviations (SD) and interquartile range (IQR). Baseline characteristics and regression equation coefficients were sampled in all analyses.

**Table 2:** Baseline characteristics (mean ± SD) of the TEMPO 3:4 study cohort [12, 24, 39]

Characteristic	Placebo arm (*N* = 484)	Treatment arm (*N* = 961)	Overall (*N* = 1445)
Male, no. (%)	251 (51.9)	495 (51.5)	746 (51.6)
Age (years)	39 ± 7	39 ± 7	38.7 ± 7.1
TKV (mL)	1668 ± 873	1705 ± 921	1692 ± 905
eGFR (mL/min/1.73 m^2^)^a^	82.14 ± 22.73	81.35 ± 21.02	81.61 ± 21.60

*CKD* chronic kidney disease, *eGFR* estimated glomerular filtration rate, *TKV* total kidney volume

^a^Baseline eGFR was based on the Chronic Kidney Disease Epidemiology Collaboration (CKD-Epi) equation [15], used to ascertain CKD stage at baseline

To assess the influence of baseline characteristics on model predictions, the ADPKD-OM was used to predict age at ESRD onset for hypothetical patient cohorts with varying baseline age (25–45 years), eGFR (60–110 mL/min/1.73 m^2^) and TKV (1000–2000 mL). Univariate sensitivity analyses were additionally performed to independently explore the impact of baseline age (± 10 years), eGFR (± 20%), TKV (± 20%) and gender (0–100% female) on predicted outcomes, using illustrative cohorts with increasingly advanced stages of ADPKD progression at baseline.

### Illustrative analysis

The ADPKD-OM was used to simulate disease progression in three real-world clinical examples: a “rapidly progressing patient” as per ERA-EDTA recommendations [8], with high TKV and low eGFR; a “young patient”, with large kidneys for their age; and an “older patient with preserved renal function”. Baseline profiles of each hypothetical cohort were defined in terms of clinical characteristics that drive prediction of disease progression in the ADPKD-OM (age, gender, TKV and eGFR), in addition to descriptive characteristics that may be associated with these predictive risk factors.

## Results

### ADPKD progression equations

Age, gender and TKV were selected as covariates to model annual changes in TKV and eGFR within the ADPKD-OM. Tables 3 and 4 present the coefficient estimates for the TKV and eGFR progression equations, respectively; associated variance covariance matrices are provided in Additional file 1 (Tables S1 and S2). An example of using the disease progression equations to calculate annual TKV growth and eGFR decline in a hypothetical ADPKD patient is also provided in Additional file 1.

**Table 3:** Coefficient estimates for the TKV progression equation (Eq. 1), as derived from TEMPO 3:4 patient-level TKV data

	Coefficient estimate	SE	*t* value	Pr(>|t|)
Intercept (λ)	0.7889	1.1313	0.697	0.4860
Age (years) (α)	0.1107	0.0287	3.858	0.0001
Ln(Baseline TKV) (β)	0.8027	0.1556	5.159	0.0000
Sex (female = 1, male = 0) (γ)	−0.0486	0.0266	−1.827	0.0684
Age:Ln(Baseline TKV) (δ)	−0.0160	0.0039	−4.058	0.0001

*SE* standard error, *TKV* total kidney volume

**Table 4:** Coefficient estimates for the eGFR progression equation (Eq. 2), as derived from TEMPO 3:4 patient-level reciprocal of serum creatinine measurements

	Coefficient estimate	SE	*t* value	Pr(>|t|)
Intercept (λ)	4.48474	0.08244	54.398	<0.0001
Ln(TKV) (β)	−0.06227	0.01124	−5.539	<0.0001

*SE* standard error, *TKV* total kidney volume

Consistent with published literature and clinical opinion, patient-level analysis of TEMPO 3:4 data supported the selection of age and baseline TKV as predictive risk factors for annual TKV growth, with positive coefficient estimates relating increased rate of TKV growth with advancing age and larger kidneys. A negative coefficient value for the interaction term between age and baseline TKV indicated that the relative impact of TKV on disease progression decreased over time. Despite not reaching a conventional level of significance (*p* = 0.0684), gender was additionally selected as a predictive risk factor of TKV growth, due to its recognised correlation with height-adjusted TKV (an alternative prognostic indicator not available in the TEMPO 3:4 dataset).

Patient-level analysis similarly supported the selection of current TKV as a predictive risk factor for annual eGFR decline, with a negative coefficient estimate relating larger kidneys to an increased rate of eGFR decline. Additional sensitivity analyses demonstrated that the impact of using CKD-Epi measurements to fit the coefficients of the eGFR progression equation was minimal (Additional file 1: Tables S3 and S4; Additional file 2: Figure S1).

### Model validation

**Fig. 2:**
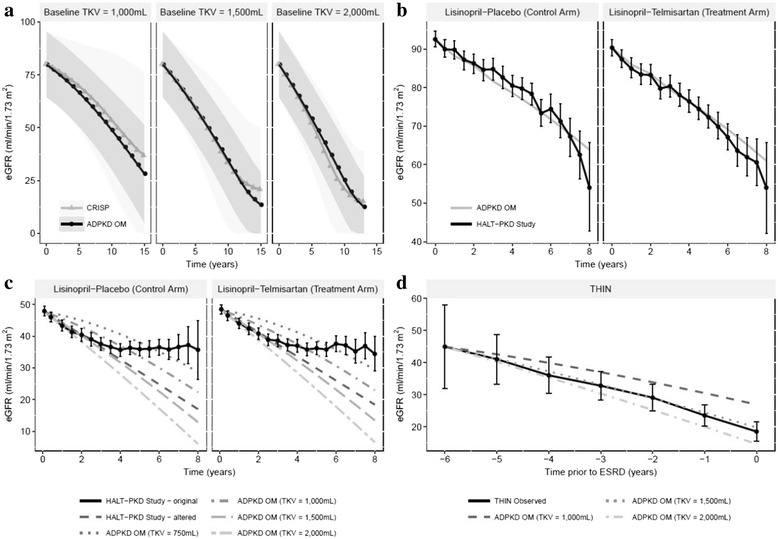
Validation of the TEMPO 3:4 disease progression equations implemented within the ADPKD Outcomes Model. Trajectories of eGFR progression were consistent with predictions derived from equations fitted to CRISP I data (panel **a**), while model-predicted eGFR progression was consistent with observed data from HALT-PKD Study A (panel **b**), HALT-PKD Study B (panel **c**), and THIN database (panel **d**). Shaded regions depict 95% prediction intervals; error bars depict 95% confidence intervals. ADPKD-OM: autosomal dominant polycystic kidney disease Outcomes Model; CRISP: Consortium for Radiologic Imaging Studies of Polycystic Kidney Disease study; eGFR: estimated glomerular filtration rate; ESRD: end-stage renal disease; HALT-PKD: Halt Progression of Polycystic Kidney Disease trials; THIN: The Health Improvement Network; TKV: total kidney volume

Predicted trajectories of disease progression from the ADPKD-OM were consistent with predictions derived from equations fitted to CRISP I data for eGFR (Fig. 2a) and TKV (Additional file 3: Figure S2). As the CRISP I study population was not fully enriched for patients with rapidly progressing ADPKD, 95% prediction intervals were found to widen over time.

Model predictions were also consistent with the results of both HALT-PKD trials. Using baseline characteristics from early-stage patients in Study A, the ADPKD-OM predicted annual eGFR (Fig. 2b) and TKV (Additional file 4: Figure S3) measurements within the 95% confidence interval of trial observations. Validation against late-stage patients in HALT-PKD Study B was complicated by the lack of TKV data and a reduction in eGFR decline toward study end, likely due to attrition of participants with rapid renal progression [20]. Nevertheless, ADPKD-OM predictions closely replicated the trial observations after adjustment for this potential survivor effect from year 4 onwards, when a baseline TKV of 1000–1500 mL was modelled (Fig. 2c). Approximate average annual slopes predicted for baseline TKV of 1000 mL and 1500 mL were −3.2 and −4.4 mL/min/1.73 m^2^, respectively, compared with −3.9 mL/min/1.73 m^2^ reported in the trial.

To further validate late-stage ADPKD patients, eGFR predictions from the ADPKD-OM were compared with observational patient-level data from the THIN database for 64 patients, over 6 years prior to ESRD. Predicted trajectories were consistent with observed data, with a modelled baseline TKV of 1500 mL achieving the best fit (Fig. 2d).

### Model application

In a simulated cohort consistent with the rapidly progressing TEMPO 3:4 population at baseline, the mean age at ESRD onset was predicted to be 52.4 years (median 53.4; IQR 47.1–60.1; SD 10.0). Prior to ESRD, modelled ADPKD patients spent a mean of 13.6 years in CKD stages 1–4 (median 13.9; IQR 10.9–17.4; SD 6.8), with 5.6, 5.4 and 2.5 years spent in CKD stages 2, 3 and 4, respectively. It was predicted that 98% of the simulated cohort would progress to ESRD over a lifetime. Most of the modelled cohort reached ESRD prior to the age many individuals retire (62–67 years across European countries), with a substantial proportion progressing earlier in life: 18, 36, 56, 73 and 85% by the age of 45, 50, 55, 60 and 65 years, respectively.

**Fig. 3:**
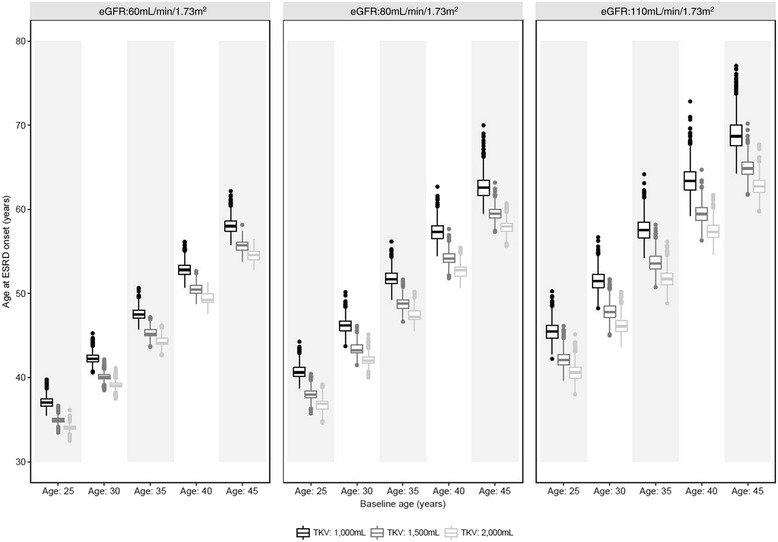
Age at ESRD onset as a function of baseline patient characteristics: eGFR (60–110 mL/min/1.73 m^2^); age (25–45 years); TKV (1000–2000 mL). Midline represents median; upper and lower hinges represent 25th and 75th percentiles; upper and lower whiskers represent highest and lowest values within 1.5 times the interquartile range; data beyond the whiskers are plotted as outliers. eGFR: estimated glomerular filtration rate; ESRD: end-stage renal disease; TKV: total kidney volume

Figure 3 demonstrates the influence of baseline patient characteristics on ADPKD-OM predictions of disease progression. Baseline age had a modest impact on age at ESRD onset among simulated cohorts with the same renal characteristics (eGFR and TKV), with patients aged 45 years at baseline reaching ESRD up to 3.1 years later than those aged 25 years. In comparison, the relationship between baseline TKV and age at ESRD was more pronounced and evident across all baseline age groups; and the relative impact of TKV on ESRD onset was similar across baseline eGFR levels. Thus, patient cohorts with larger kidneys at a younger age were predicted to progress to ESRD at an earlier age, with less variation in predicted outcomes.

**Fig. 4:**
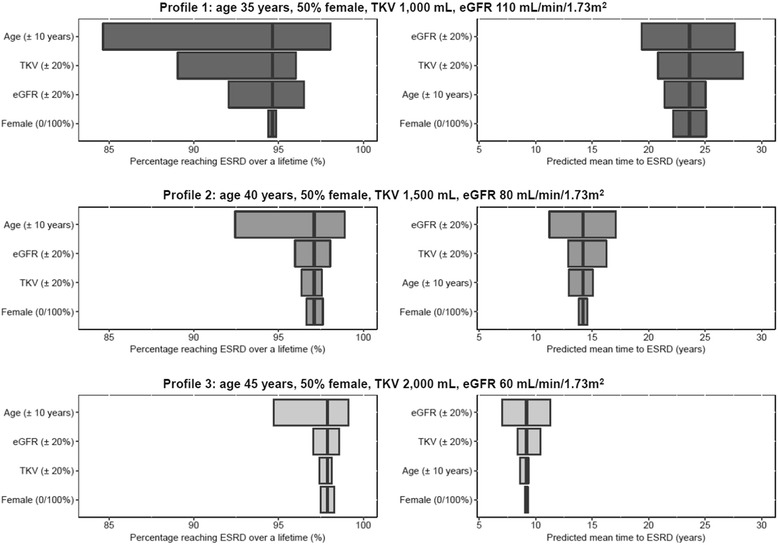
Univariate sensitivity analyses demonstrating the influence of baseline patient characteristics on lifetime ESRD risk and predicted age at ESRD onset. Profiles 1–3 represent hypothetical patient cohorts with increasingly advanced stages of ADPKD progression at baseline. Vertical lines represent base-case values. eGFR: estimated glomerular filtration rate; ESRD: end-stage renal disease; TKV: total kidney volume

The results of univariate sensitivity analyses comparing the influence of baseline patient characteristics on predicted long-term outcomes are displayed in Fig. 4. As cohorts with increasingly advanced ADPKD were simulated, the proportion of patients expected to reach ESRD increased and the mean predicted time to ESRD onset decreased. Baseline eGFR was a driver of predicted outcomes, associated with up to 4% difference in the probability of lifetime ESRD incidence and up to 8 years' difference in the time to ESRD onset. However, the likelihood of ESRD was most sensitive to baseline age (up to 13% difference), and the mean time to ESRD onset was also influenced by baseline TKV (up to 7 years’ difference). By contrast, gender had a comparatively small effect on predicted outcomes. Across the characteristics examined, the variation in predicted outcomes consistently decreased with increasingly advanced disease characteristics at baseline.

### Illustrative analysis

**Fig. 5:**
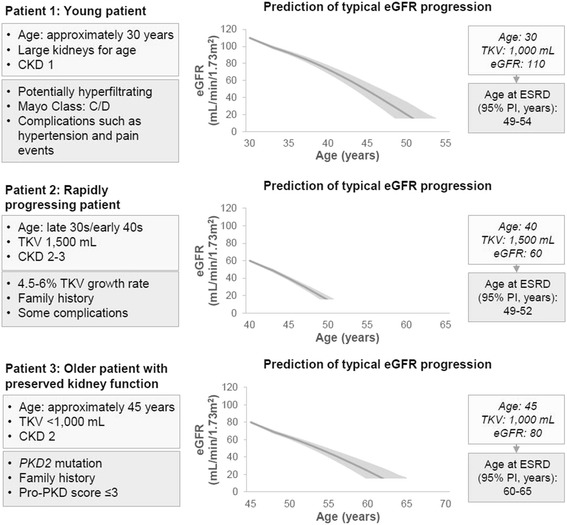
Prediction of disease progression for three illustrative ADPKD patient profiles. Upper sections of patient characteristics describe modelled drivers of progression. CKD: chronic kidney disease; eGFR: estimated glomerular filtration rate; ESRD: end-stage renal disease; TKV: total kidney volume; PI: prediction interval

Figure 5 illustrates ADPKD-OM predictions of disease progression across three real-world clinical examples. The model predicted that a “rapidly progressing patient”, with high TKV and low eGFR, would progress to ESRD at 49–52 years of age. A “young patient” with large kidneys for their age was predicted to reach ESRD at age 49–54 years, in contrast to an “older patient with preserved kidney function” predicted to progress to ESRD at age 60–65 years. Relative to the other patient profiles, the truncated time to ESRD shown by the “rapidly progressing patient” illustrates the importance of TKV as a predictor of disease progression.

## Discussion

At present ADPKD is an incurable condition, with patients experiencing heterogeneous rates of progression towards kidney failure over several decades. As targeted therapies are developed, there is an increased need for user-friendly tools that accurately predict the natural history of ADPKD, and distinguish rapidly progressing patients most likely to benefit from treatment. To facilitate the timely identification of patients with greatest unmet need, the present study sought to develop a simulation model capable of predicting the long-term risk and rate of progression towards ESRD, where inputs are limited to readily observable and/or measurable characteristics of ADPKD patients. After implementing progression equations derived from available TEMPO 3:4 trial data, disease progression estimates generated by the ADPKD-OM were assessed for clinical plausibility and validated against external data sources. In combination with clinical judgement, the ADPKD-OM subsequently represents a valuable resource to predict the natural history of ADPKD for research and clinical practice.

The ADPKD-OM generates accurate predictions of disease progression and long-term outcomes for hypothetical cohorts, with respect to clinical characteristics that are readily available and/or measurable among those with ADPKD. Covariates to inform the development of TKV and eGFR progression equations were evaluated based on predictive factors identified in the literature [1, 3, 8–10, 12, 18, 22] and the opinions of clinical experts in ADPKD management, and were selected in conjunction with patient-level analysis of the TEMPO 3:4 placebo arm. Our analyses supported the selection of age and baseline TKV as strong predictive risk factors for annual TKV growth, and current TKV as a powerful predictive risk factor for future rate of eGFR decline. The inclusion of gender as an additional predictor of TKV growth is consistent with general statistical guidance, in which variables known to be relevant are retained despite not achieving a conventional level of significance [23].

In the absence of a lifetime trial or registry of ADPKD patients, the TEMPO 3:4 trial that informed the development of ADPKD-OM progression equations remains to be one of the largest sources of high-quality patient-level data in this field [12, 24]. The TEMPO 3:4 study population comprised of ADPKD patients aged ≤50 years, with TKV ≥750 mL and an estimated creatine clearance of ≥60 mL/mL; therefore, by design, TEMPO 3:4 selected for patients with early-stage disease and preserved renal function, but with high likelihood of rapid progression. Since model validity is dependent on the data used to inform its development, caution is required when simulating populations that do not conform to the TEMPO 3:4 cohort profile. However, external validation exercises demonstrated that ADPKD-OM predictions of disease progression were credible when alternative populations, not enriched for patients with rapid progression, were simulated. Modelled trajectories of eGFR and TKV in early-stage ADPKD patients were validated against data derived from CRISP I [18] and HALT-PKD Study A [19], while model-predicted disease progression in late-stage disease was consistent with observations from HALT-PKD Study B [20] and THIN [21]. Furthermore, the ADPKD-OM was developed in line with National Institute for Health and Care Excellence (NICE) Decision Support Unit guidance [25] and was designed, constructed and tested according to good practice guidelines [26]. Model predictions have undergone independent scrutiny as part of health technology assessments carried out by NICE and the Scottish Medicines Consortium, and were considered sufficiently robust to inform health economic decision-making in ADPKD [27, 28]. As new data in this field becomes available, ongoing validation and refinement of the ADPKD-OM will ensure the continued accuracy of model predictions, and extend its generalisability to other ADPKD populations in the future.

Simulation of hypothetical patient cohorts within the validated ADPKD-OM highlighted the burden associated with ADPKD, and its progression towards ESRD, on society and public healthcare systems. In a cohort consistent with the TEMPO 3:4 population at baseline, the predicted mean age at ESRD onset was 52 years, with the majority of patients having progressed to ESRD prior to retirement age (62–67 years across European countries) [29]. Differences in the mean age at ESRD predicted by the model compared with estimates in the literature [2, 5, 6, 10, 22] are reflective of the rapidly progressing patient population selected as a result of TEMPO 3:4 eligibility criteria [12, 24]. However, modelling disease progression in alternative, real-world clinical examples similarly demonstrated significant ESRD risk over the course of a patient’s lifetime. This study presents the ADPKD-OM as a validated approach to simulate the natural progression of ADPKD and predict long-term public health outcomes in the absence of therapy; an important feature to underpin future applications of the model. Extending the ADPKD-OM beyond ESRD onset will enhance the model’s ability to evaluate fully the long-term burden of ADPKD, predict patient life expectancy, and quantify outcomes associated with RRT. Furthermore, the incorporation of costs and treatment effects will allow the health economic value of future therapeutic management practices to be estimated in the absence of long-term clinical evidence.

This study additionally highlighted the influence of baseline patient characteristics on ADPKD-OM predictions of disease progression. Analyses demonstrated the sensitivity of predicted ADPKD progression to baseline age, TKV and eGFR, and the value of TKV as an early prognostic factor for future ESRD risk; findings that are aligned with those of published literature [3, 10, 18]. Such research has informed the development of the Mayo classification of ADPKD [9]; and recommendations issued by the EMA and the US Food and Drug Administration, which collectively qualify TKV, in combination with patient age and baseline eGFR, as a prognostic biomarker for patient selection in clinical trials of ADPKD [30, 31]. In clinical practice, the ERA-EDTA consensus statement similarly recognises the value of documented eGFR decline, documented TKV growth and patient age, in addition to factors such as CKD stage, ADPKD genotype and family history, to assess the risk of rapid progression among ADPKD patients [8]. While the ERA-EDTA algorithm and Mayo classification system are valuable tools to evaluate a patient’s likelihood of rapid progression and eligibility for treatment in clinical research and practice, the ADPKD-OM provides an additional resource that communicates long-term risk in terms of predicted age at ESRD onset.

Although the ADPKD-OM simulates progression towards ESRD with respect to established drivers of baseline TKV, eGFR, patient age and gender, it does not consider additional factors for which data was not captured during TEMPO 3:4. Consistent with the approach of other ADPKD progression models [9, 22], the ADPKD-OM did not account for hypertension and proteinuria, as the clinical presentation of these factors is heterogeneous across patients [32, 33]. Similarly, the consequences of extra-renal manifestations, including polycystic liver disease or cardiovascular disease, were not modelled due to a lack of published data on the prevalence of ADPKD-related complications. The model does not consider differences in ADPKD progression due to *PKD1* or *PKD2* mutation [34, 35]; however, since genotype is a major determinant of baseline TKV [1, 36, 37], the inclusion of TKV within the model may adequately account for this limitation. Furthermore, mortality risk in ADPKD patients prior to ESRD was conservatively assumed to be consistent with that reported for the general UK population, due to a lack of ADPKD-specific mortality data. While this approach may underestimate mortality and consequently overestimate the percentage of patients expected to reach ESRD in ADPKD-OM simulations, predictions of time to ESRD among patients who progress would not be unduly biased.

Trajectories of disease progression generated by the ADPKD-OM should be interpreted with its limitations in mind, and should not replace clinical judgement. However, in conjunction with clinical assessment and other predictive algorithms, the ADPKD-OM represents an informative tool to assess risks and long-term outcomes in ADPKD patients; and support decision-making in research and clinical practice. By simulating hypothetical patient cohorts of varying age, eGFR and TKV, this study demonstrated the ability of the ADPKD-OM to accurately predict long-term disease progression towards ESRD as a function of observable and/or measurable patient characteristics. While eGFR and clinical experience of symptoms are traditionally used to predict disease progression in clinical assessment, the measurement of renal volume in ADPKD patients is not yet considered routine practice. Despite this, validation exercises found that in the absence of TKV data, disease progression predicted by the ADPKD-OM remained plausible over a range of baseline values. As the prognostic value of TKV growth in ADPKD patients is increasingly recognised and advocated [8, 38], the adoption of this measure in routine clinical practice will only enhance predictions of disease progression generated by the ADPKD-OM.

## Conclusions

This study developed a natural history model of ADPKD, which serves to predict disease progression and long-term outcomes with respect to characteristics that are readily observable and/or measurable among ADPKD patients. Simulation of hypothetical cohorts within the ADPKD-OM demonstrated the model’s validity when compared against external data sources; and the sensitivity of its predictions to baseline age, TKV and eGFR. Irrespective of patient characteristics, predictions of disease progression in real-world clinical examples highlighted the potential long-term burden of ADPKD to society and public healthcare systems. At present ADPKD is an incurable condition that exhibits heterogeneous rates of disease progression towards ESRD; however, the ADPKD-OM represents a valuable tool that utilises available clinical data to identify patients most likely to benefit from novel therapies in research and clinical practice.

## Additional files

Additional file 1: Variance covariance matrices for TKV and eGFR progression equation coefficients. **Table S1.** Variance covariance matrix for the TEMPO 3:4 TKV equation coefficients. **Table S2.** Variance covariance matrix for the TEMPO 3:4 eGFR equation coefficients. Example of using the TKV and eGFR progression equations to predict annual ADPKD progression. Applying the ADPKD-OM to alternative patient populations. Using CKD-Epi measurements to model eGFR progression. **Table S3.** Comparison of eGFR progression equation coefficient estimates. **Table S4.** Variance covariance matrix for the TEMPO 3:4 eGFR equation coefficients. Validation against CRISP I-derived progression equations. CRISP I-derived equations for TKV (**Equation S1**) and eGFR (**Equation S2**) progression. **Table S5.** TKV progression equation coefficient estimates, as derived from CRISP I. **Table S6.** eGFR progression equation coefficient estimates, as derived from CRISP I. Validation against HALT-PKD trial data. Validation against THIN data. Validation against Thong and Ong [40]. **Equation S3.** eGFR progression equation, derived by Thong and Ong [40]. **Table S7.** eGFR progression equation coefficient estimates, as derived from Thong and Ong [40]. (DOCX 51 kb)

Additional file 2: Figure S1. Illustrative eGFR trajectories predicted using coefficient estimates derived from the reciprocal of serum creatinine or CKD-Epi measurements. 1/SC: reciprocal of serum creatinine CKD-Epi: Chronic Kidney Disease Epidemiology Collaboration; eGFR: estimated glomerular filtration rate. (JPEG 50 kb)

Additional file 3: Figure S2. Comparison of TKV progression, as predicted by equations fitted to TEMPO 3:4 and CRISP I data (shaded regions depict 95% prediction intervals). Baseline patient profile: age 40 years; eGFR 80 mL/min/1.73 m^2^; TKV 1000, 1500 and 2000 mL; 48.4% female. ADPKD-OM: autosomal dominant polycystic kidney disease Outcomes Model; CRISP: Consortium for Radiologic Imaging Studies of Polycystic Kidney Disease study; TKV: total kidney volume. (JPEG 267 kb)

Additional file 4: Figure S3. Validation of the TEMPO 3:4 disease progression equations implemented within the ADPKD Outcomes Model. Trajectories of TKV progression were consistent with observed data from HALT-PKD Study A. Error bars depict 95% confidence intervals. ADPKD-OM: autosomal dominant polycystic kidney disease Outcomes Model; HALT-PKD: Halt Progression of Polycystic Kidney Disease trials; TKV: total kidney volume. (JPEG 58 kb)

## Abbreviations

ADPKD: Autosomal dominant polycystic kidney disease
ADPKD-OM: Autosomal dominant polycystic kidney disease Outcomes Model
CKD: Chronic kidney disease
CKD-Epi: Chronic Kidney Disease Epidemiology Collaboration
CRISP: Consortium for Radiologic Imaging Studies of Polycystic Kidney Disease study
eGFR: estimated glomerular filtration rate
EMA: European Medicines Agency
ERA-EDTA: European Renal Association-European Dialysis and Transplantation Association
ESRD: End-stage renal disease
GFR: Glomerular filtration rate
HALT-PKD: Halt Progression of Polycystic Kidney Disease trials
IQR: Interquartile range
NICE: National Institute for Health and Care Excellence
RRT: Renal replacement therapy
SD: Standard deviation
TEMPO 3:4: Tolvaptan Efficacy and Safety in Management of Autosomal Dominant Polycystic Kidney Disease and its Outcomes 3:4 Study
THIN: The Health Improvement Network
TKV: Total kidney volume
