# Supplementary material for: A model to predict disease progression in patients with autosomal dominant polycystic kidney disease (ADPKD): the ADPKD Outcomes Model
Source: BMC Nephrol. 2018 Feb 13;19:37. doi: 10.1186/s12882-017-0804-2 (PMC5810027; doi:10.1186/s12882-017-0804-2)
Supplement: Supplementary file 1 — Variance covariance matrices for TKV and eGFR progression equation coefficients. Table S1. Variance covariance matrix for the TEMPO 3:4 TKV equation coefficients. Table S2. Variance covariance matrix for the TEMPO 3:4 eGFR equation coefficients. Example of using the TKV and eGFR progression equations to predict annual ADPKD progression. Applying the ADPKD-OM to alternative patient populations. Using CKD-Epi measurements to model eGFR progression. Table S3. Comparison of eGFR progression equation coefficient estimates. Table S4. Variance covariance matrix for the TEMPO 3:4 eGFR equation coefficients. Validation against CRISP I-derived progression equations. CRISP I-derived equations for TKV (Equation S1) and eGFR (Equation S2) progression. Table S5. TKV progression equation coefficient estimates, as derived from CRISP I. Table S6. eGFR progression equation coefficient estimates, as derived from CRISP I. Validation against HALT-PKD trial data. Validation against THIN data. Validation against Thong and Ong [40]. Equation S3. eGFR progression equation, derived by Thong and Ong [40]. Table S7. eGFR progression equation coefficient estimates, as derived from Thong and Ong [40]. (DOCX 51 kb) [file 12882_2017_804_MOESM1_ESM.docx]

# Additional file 1

# Variance covariance matrices

Variance covariance matrices for TKV and eGFR equation coefficient estimates are detailed in Table S1 and Table S2, respectively; associated coefficient estimates are provided in McEwan et al (Table 3 and Table 4).

Table S1 Variance covariance matrix for the TEMPO 3:4 TKV equation coefficients.

| **Covariance** | **Intercept** | **Age (years)** | **Ln(TKV)** | **Female** | **Age:Ln(TKV)** |
| --- | --- | --- | --- | --- | --- |
| **Intercept** | 1.279758 | -0.031790 | -0.175654 | -0.001306 | 0.004362 |
| **Age (years)** | -0.031790 | 0.000823 | 0.004361 | -0.000016 | -0.000113 |
| **Ln(TKV)** | -0.175654 | 0.004361 | 0.024207 | 0.000155 | -0.000601 |
| **Female** | -0.001306 | -0.000016 | 0.000155 | 0.000708 | 0.000002 |
| **Age:Ln(TKV)** | 0.004362 | -0.000113 | -0.000601 | 0.000002 | 0.000016 |
| *TKV: total kidney volume.* | | | | | |

Table S2 Variance covariance matrix for the TEMPO 3:4 eGFR equation coefficients.

| **Covariance** | **Intercept** | **Ln(TKV)** |
| --- | --- | --- |
| **Intercept** | 0.006797 | -0.000925 |
| **Ln(TKV)** | -0.000925 | 0.000126 |
| *TKV: total kidney volume.* | | |

# Using the TKV and eGFR progression equations to predict annual ADPKD progression

For a 30-year-old female, with current TKV of 1,000 mL and eGFR of 110 mL/min/1.73 m^2^, predicted one-year ADPKD progression is calculated as:

*TKV change = exp[0.7889 + (0.1107 x 30) + (0.8027 x ln(1,000) - 0.0486 - (0.0160 x 30 x ln(1,000))] - 500 = +39 mL*

*eGFR change = exp[4.48474 - (0.06227 x ln(1,000))] - 60 = -2.3 ml/min/1.73m^2^*

# Applying the ADPKD-OM to alternative patient populations

Patients eligible for enrollment in TEMPO 3:4 were aged ≤50 years with TKV ≥750 mL at baseline. Furthermore, a limited number of patients with baseline TKV of 750-850 mL were enrolled in the trial. To simulate disease progression in cohorts that do not conform to the TEMPO 3:4 patient profile, regression equations within the ADPKD-OM required the following limits to be applied:

- For patients with baseline TKV <850 mL, the equation utilised a value of 850 mL.
- For modelled patients >50 years of age, annual change in TKV was assumed equal to the annual change at 50 years of age.

# Using CKD-Epi measurements to model eGFR progression

Consistent with National Institute for Health and Care Excellence (NICE) guidelines [1], estimated glomerular filtration rate (eGFR) was alternatively measured in TEMPO 3:4 using the Chronic Kidney Disease Epidemiology Collaboration (CKD-Epi) equation [2]. When this method was applied, mean (±SD) baseline eGFR in the placebo arm of TEMPO 3:4 was 82.14±22.73 mL/min/1.73m^2­­­^, and mean annual change in eGFR was -3.568±4.495 mL/min/1.73m^2­­­^ [3, 4]. CKD-Epi measurements were used to fit the coefficients of the eGFR progression equation (Equation 2 in McEwan et al), and compared against those derived from the reciprocal of serum creatinine data. Regression coefficient estimates for each approach are provided in Table S3; the variance covariance matrix for these coefficient estimates is detailed in Table S4. When compared to the eGFR trajectory modelled using the reciprocal of serum creatinine data, the impact of using CKD-Epi measurements to fit the coefficients of the eGFR progression equation was minimal (Figure S1).

Table S3 Comparison of eGFR progression equation coefficient estimates.

|  | **Coefficient estimate** | **SE** | ***t* value** | **Pr(>\|t\|)** |
| --- | --- | --- | --- | --- |
| **1/SC** |  |  |  |  |
| Intercept (λ) | 4.48474 | 0.08244 | 54.398 | <2e-16 |
| Ln(TKV) (β) | -0.06227 | 0.01124 | -5.539 | 5.17e-08 |
| **CKD-Epi** |  |  |  |  |
| Intercept (λ) | 4.46867 | 0.07616 | 58.672 | < 2e-16 |
| Ln(TKV) (β) | −0.06002 | 0.01039 | -5.779 | 1.4E-08 |
| *1/SC: reciprocal of serum creatinine; CKD-Epi: Chronic Kidney Disease Epidemiology Collaboration; SE: standard error; TKV: total kidney volume.* | | | | |

Table S4 Variance covariance matrix for the TEMPO 3:4 eGFR equation coefficients.

| **Covariance** | **1/SC** | | **CKD-Epi** | |
| --- | --- | --- | --- | --- |
|  | **Intercept** | **Ln(TKV)** | **Intercept** | **Ln(TKV)** |
| **Intercept** | 0.006797 | -0.000925 | 0.005801 | -0.000790 |
| **Ln(TKV)** | -0.000925 | 0.000126 | -0.000790 | 0.000108 |
| *1/SC: reciprocal of serum creatinine; CKD-Epi: Chronic Kidney Disease Epidemiology Collaboration; TKV: total kidney volume.* | | | | |

# Validation sources

To establish the accuracy with which the ADPKD-OM reproduces the natural history of ADPKD and investigate the generalisability of its predictions to other populations, external validation exercises were performed using multiple data sources. Predictions were compared with observed data derived from the following studies, which included patient-level or aggregate data for different stages of ADPKD and in differing settings.

1. **CRISP I-derived progression equations**

The Consortium for Radiologic Imaging Studies of Polycystic Kidney Disease (CRISP) was an observational study of ADPKD patients that used high-resolution magnetic resonance imaging (MRI) to determine if changes in renal and cyst volumes could be detected over a short period of time; and whether they correlated with a decline in renal function early in disease [5]. Observations from 3-year (CRISP I) [6] and 8-year studies (CRISP II) [7] demonstrated that TKV and eGFR are associated with the rate of ADPKD progression.

Regression equations were fitted to annual TKV and eGFR changes observed in aggregated CRISP I data, stratified by age (aged <30 or ≥30 years) and TKV (TKV <750 mL or ≥750 mL; and TKV <1,500 mL or ≥1,500 mL) [6], such that all simulated individuals in each age and TKV category experienced the same change in eGFR and TKV. Equations for TKV and eGFR progression derived from CRISP I are provided in Equation S1 and Equation S2; coefficient estimates for each are provided in Table S5 and Table S6, respectively. Trajectories of disease progression using TEMPO 3:4 progression equations were found to be consistent with predictions derived from equations fitted to CRISP I data for eGFR (Figure 2A, McEwan et al) and TKV at lower values (Figure S2).

**Equation S1: TKV_t+1_ = TKV_t_ + exp(λ + β.TKV_t_)**

*TKV, total kidney volume; t, time; β, TKV coefficient; λ, intercept.*

**Equation S2: eGFR_t+1_ = eGFR_t_ + λ + β.ln(TKV_t_)**

*eGFR, estimated glomerular filtration rate; t, time; β, TKV coefficient; λ, intercept.*

Table S5 TKV progression equation coefficient estimates, as derived from CRISP I.

|  | **TKV coefficient (β)** | | **Intercept (λ)** | |
| --- | --- | --- | --- | --- |
|  | **Estimate** | **SE** | **Estimate** | **SE** |
| Age <30 years | 0.00139 | 0.00005 | 2.57423 | 0.06310 |
| Age ≥30 years | 0.00111 | 0.00022 | 2.64441 | 0.31877 |
| *SE: standard error; TKV: total kidney volume* | | | | |

Table S6 eGFR progression equation coefficient estimates, as derived from CRISP I.

|  | **Ln(TKV) coefficient (β)** | | **Intercept (λ)** | |
| --- | --- | --- | --- | --- |
|  | **Estimate** | **SE** | **Estimate** | **SE** |
| Age <30 years | -4.28336 | 0.38979 | 29.40597 | 2.68969 |
| Age ≥30 years | -4.58191 | 0.12019 | 30.17095 | 0.84317 |
| *eGFR: estimated glomerular filtration rate; SE: standard error; TKV: total kidney volume.* | | | | |

1. **HALT-PKD trial**

The Halt Progression of Polycystic Kidney Disease (HALT-PKD) trial was a double-blind, placebo-controlled trial in which patients were randomly assigned to either standard or low blood-pressure targets; and to an angiotensin-converting enzyme inhibitor (lisinopril) plus an angiotensin receptor blocker (telmisartan), or lisinopril plus placebo. Study A included 558 young hypertensive participants with ADPKD (15 to 49 years of age) who had relatively preserved kidney function (eGFR >60 mL/min/1.73 m^2^) [8]. Study B included 486 older hypertensive participants with ADPKD (18 to 64 years of age) who had reduced kidney function (eGFR 25–60 mL/min/1.73 m^2^, CKD stage 3) [9].

Trajectories of eGFR predicted by TEMPO 3:4 equations within the ADPKD-OM were consistent with observed data from both HALT-PKD Study A and Study B (Figure 2 in McEwan et al). Similarly, when baseline characteristics from early-stage patients in Study A were simulated, the ADPKD-OM predicted annual TKV measurements within the 95% confidence interval of trial observations (Figure S3).

1. THIN

The Health Improvement Network (THIN) is a large primary-care database collated from general practitioners across the UK for use in research in many therapy areas, including ADPKD [10]. THIN contains longitudinal healthcare records from 570 primary-care practices with 11.7 million patients, of whom over 3.7 million are actively registered. Anonymised patient-level data for age, gender and eGFR measurements were utilised in this study; however, TKV measurements were not available.

To validate the TEMPO 3:4 progression equations in patients with late-stage disease observed in clinical practice, eGFR measurements in 64 patients prior to end-stage renal disease (ESRD) were compared with ADPKD-OM predictions for a modelled cohort with matched mean age, gender, and mean eGFR at 6 years prior to ESRD. Predicted eGFR trajectories were consistent with observed data, with a modelled baseline TKV of 1,500 mL achieving the best fit (Figure 2D in McEwan et al).

1. **Thong and Ong, 2013**

Thong and Ong conducted a retrospective analysis of ADPKD patients at a single center, based on recorded renal function up to 30 years prior to analysis [11]. The rate of eGFR decline was determined by linear regression, based on ≥5 years of renal function data prior to the study or renal replacement therapy. The published regression equation required mean kidney length and age at diagnosis as predictive variables (Equation S3); coefficient estimates are provided in Table S7.

**Equation S3: eGFR_t+1_ = eGFR_t_ + λ + β.MKL + α.AD**

*AD, age at ADPKD diagnosis; eGFR, estimated glomerular filtration rate; MKL, mean kidney length (cm); t, time; α, age at ADPKD diagnosis coefficient; β, kidney length coefficient; λ, intercept.*

**Table S7** eGFR progression equation coefficient estimates, as derived from Thong and Ong.

| **Kidney length coefficient (β)** | **Age at ADPKD diagnosis coefficient (α)** | **Intercept (λ)** |
| --- | --- | --- |
| 0.112 | -0.048 | 2.054 |
| *ADPKD: autosomal dominant polycystic kidney disease.* | | |

Since the ADPKD-OM requires baseline TKV as a variable, validation exercises using these data was not possible. However, trajectories predicted using the TEMPO 3:4 progression equations were compared with the renal progression rates predicted using the Thong and Ong regression equation. Model-predicted rates of eGFR decline and age at ESRD onset were consistent with those published from this study.

# References

1. National Institute for Health and Care Excellence. Chronic kidney disease in adults: assessment and management. Clinical guideline [CG182]. 2015. https://www.nice.org.uk/guidance/cg182. Accessed 30 March 2017.

2. Levey AS, Stevens LA, Schmid CH, Zhang YL, Castro AF, Feldman HI, et al. A new equation to estimate glomerular filtration rate. Ann Intern Med. 2009;150:604-12.

3. Torres VE, Chapman AB, Devuyst O, Gansevoort RT, Grantham JJ, Higashihara E, et al. Tolvaptan in patients with autosomal dominant polycystic kidney disease. N Engl J Med. 2012;367:2407-18.

4. Otsuka Pharmaceutical Development and Commercialization Inc. Amended Clinical Study Report. Protocol No. 156-04-251. A phase 3, multi-center, double-blind, placebo-controlled, parallel-arm trial to determine long-term safety and efficacy of oral tolvaptan tablet regimens in adult subjects with autosomal dominant polycystic kidney disease. 2013.

5. Chapman AB, Guay-Woodford LM, Grantham JJ, Torres VE, Bae KT, Baumgarten DA, et al. Renal structure in early autosomal-dominant polycystic kidney disease (ADPKD): The Consortium for Radiologic Imaging Studies of Polycystic Kidney Disease (CRISP) cohort. Kidney Int. 2003;64:1035-45.

6. Grantham JJ, Torres VE, Chapman AB, Guay-Woodford LM, Bae KT, King Jr BF, et al. Volume progression in polycystic kidney disease. N Engl J Med. 2006;354:2122-30.

7. Chapman AB, Bost JE, Torres VE, Guay-Woodford L, Bae KT, Landsittel D, et al. Kidney volume and functional outcomes in autosomal dominant polycystic kidney disease. Clin J Am Soc Nephrol. 2012;7:479-86.

8. Schrier RW, Abebe KZ, Perrone RD, Torres VE, Braun WE, Steinman TI, et al. Blood pressure in early autosomal dominant polycystic kidney disease. N Engl J Med. 2014;371:2255-66.

9. Torres VE, Abebe KZ, Chapman AB, Schrier RW, Braun WE, Steinman TI, et al. Angiotensin blockade in late autosomal dominant polycystic kidney disease. N Engl J Med. 2014;371:2267-76.

10. In Practice Systems. The Health Improvement Network (THIN). 2017. http://www.inps4.co.uk/vision/health-improvement-network-thin. Accessed 22 March 2017.

11. Thong K, Ong A. The natural history of autosomal dominant polycystic kidney disease: 30-year experience from a single centre. QJM. 2013;106:639-46.
