# Supplementary figures and images for: A model to predict disease progression in patients with autosomal dominant polycystic kidney disease (ADPKD): the ADPKD Outcomes Model
Source: BMC Nephrol. 2018 Feb 13;19:37. doi: 10.1186/s12882-017-0804-2 (PMC5810027; doi:10.1186/s12882-017-0804-2)

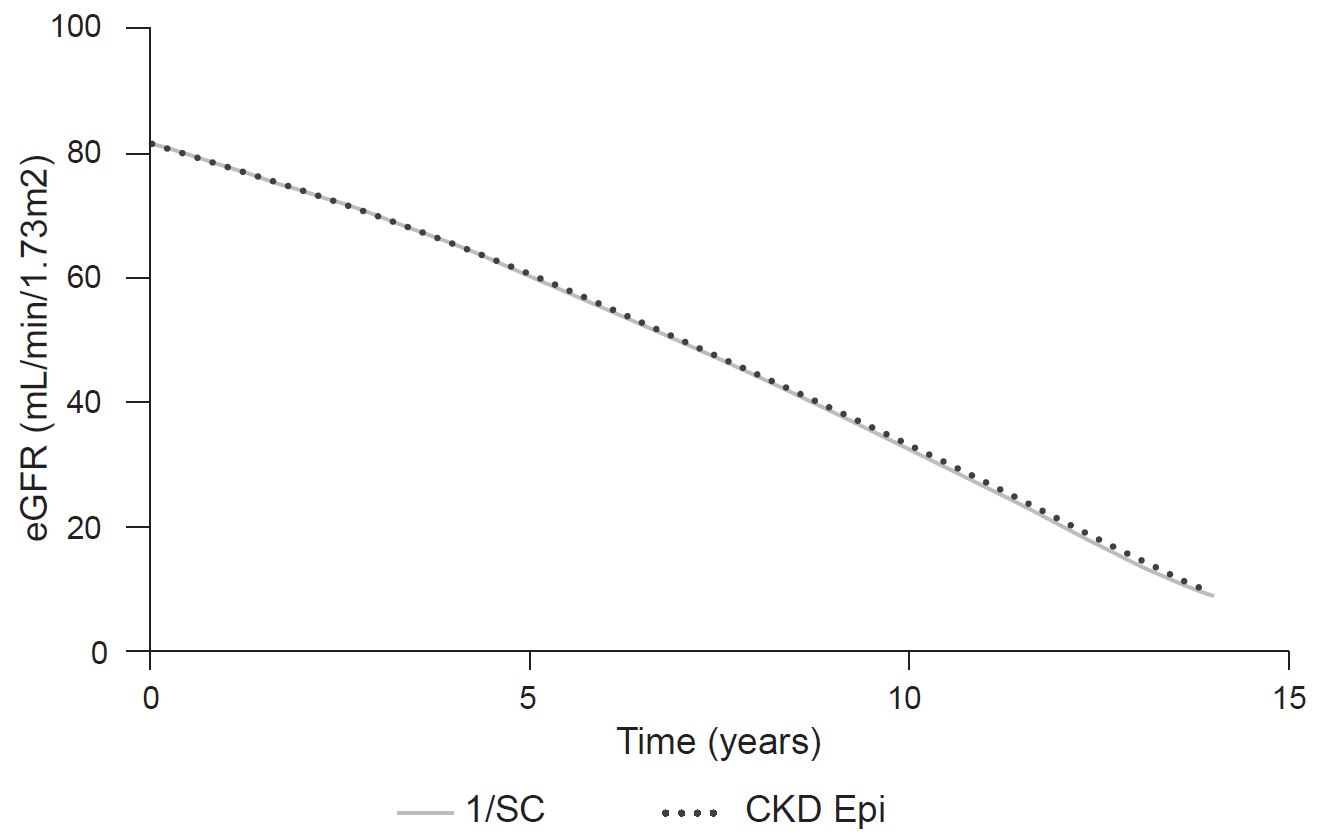

Supplement: Supplementary file 2 — Illustrative eGFR trajectories predicted using coefficient estimates derived from the reciprocal of serum creatinine or CKD-Epi measurements. 1/SC: reciprocal of serum creatinine CKD-Epi: Chronic Kidney Disease Epidemiology Collaboration; eGFR: estimated glomerular filtration rate. (JPEG 50 kb) [file 12882_2017_804_MOESM2_ESM.jpg]

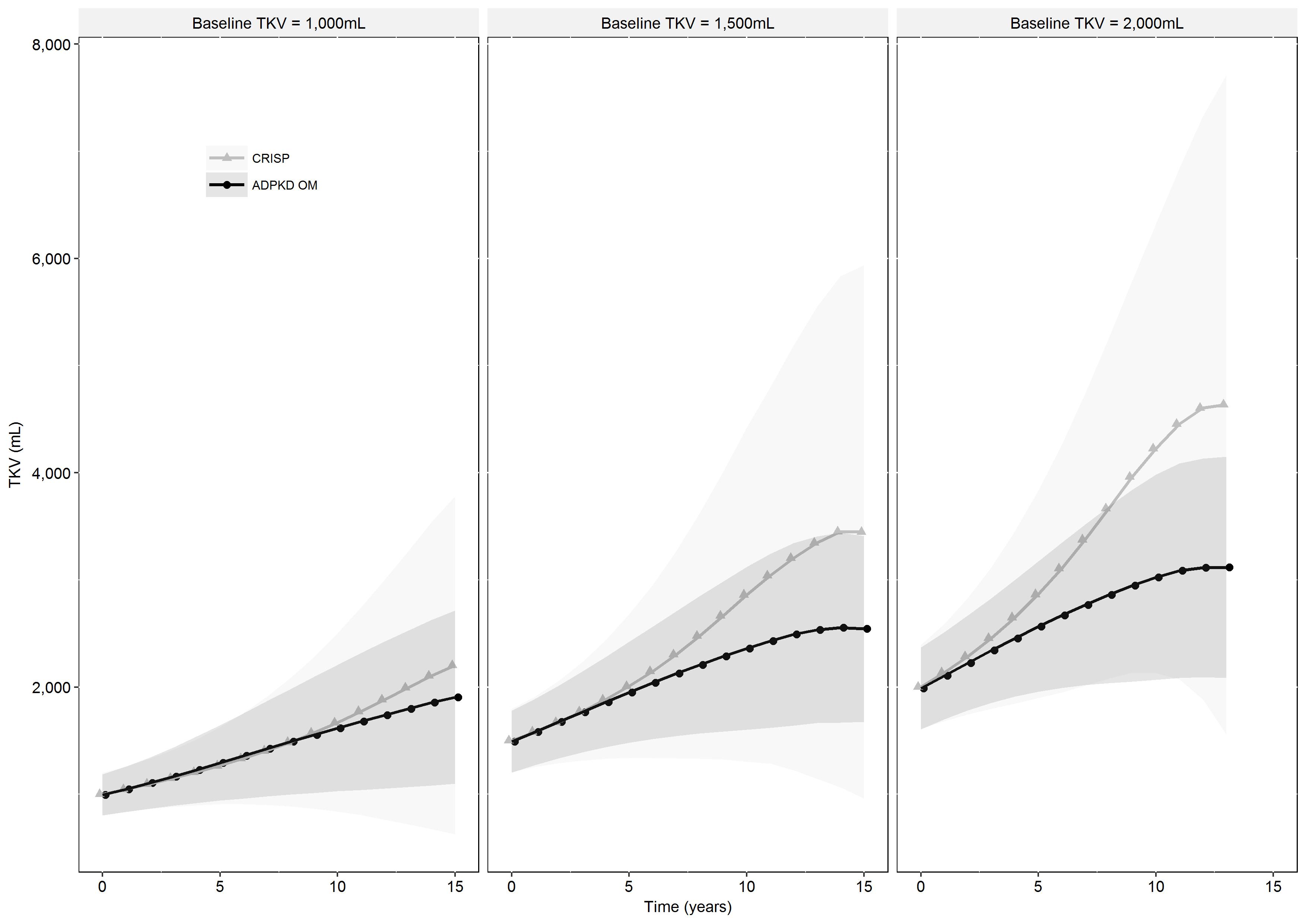

Supplement: Supplementary file 3 — Comparison of TKV progression, as predicted by equations fitted to TEMPO 3:4 and CRISP I data (shaded regions depict 95% prediction intervals). Baseline patient profile: age 40 years; eGFR 80 mL/min/1.73 m2; TKV 1000, 1500 and 2000 mL; 48.4% female. ADPKD-OM: autosomal dominant polycystic kidney disease Outcomes Model; CRISP: Consortium for Radiologic Imaging Studies of Polycystic Kidney Disease study; TKV: total kidney volume. (JPEG 267 kb) [file 12882_2017_804_MOESM3_ESM.jpg]

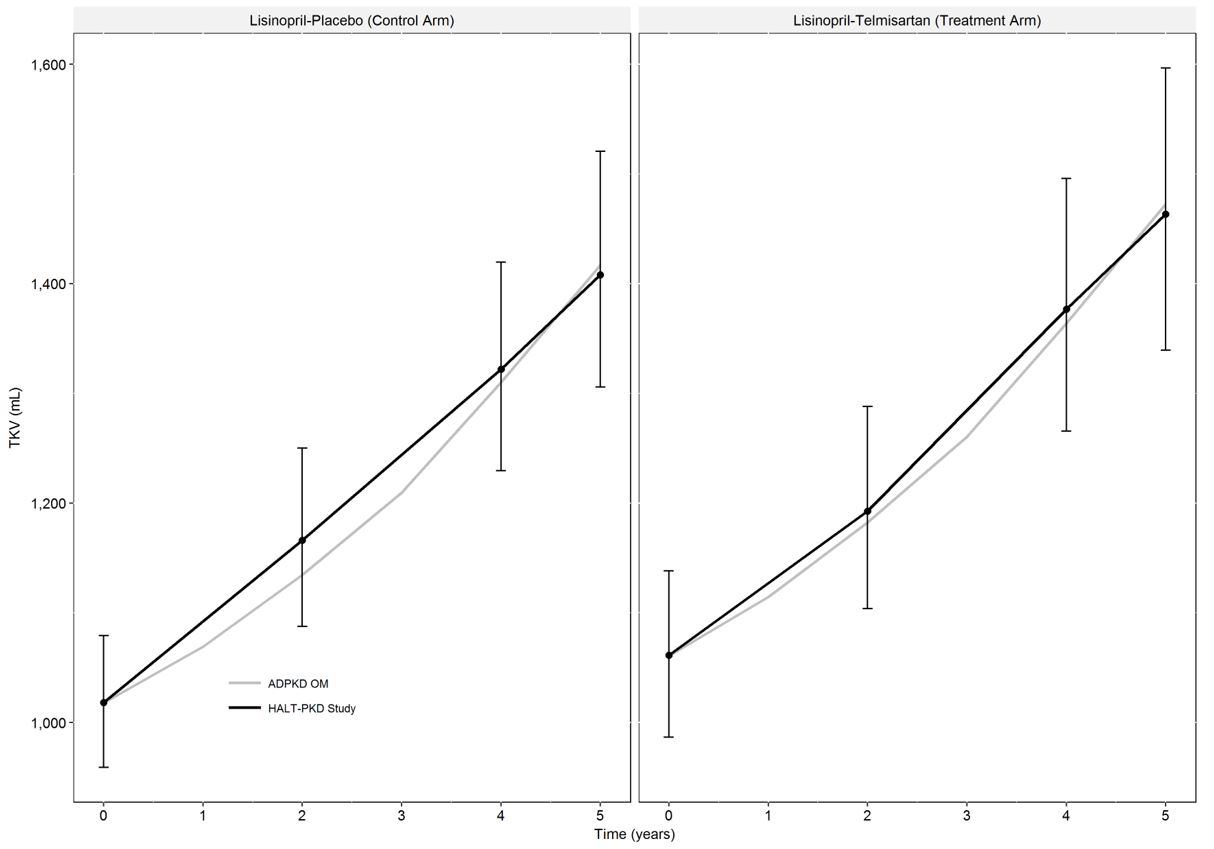

Supplement: Supplementary file 4 — Validation of the TEMPO 3:4 disease progression equations implemented within the ADPKD Outcomes Model. Trajectories of TKV progression were consistent with observed data from HALT-PKD Study A. Error bars depict 95% confidence intervals. ADPKD-OM: autosomal dominant polycystic kidney disease Outcomes Model; HALT-PKD: Halt Progression of Polycystic Kidney Disease trials; TKV: total kidney volume. (JPEG 58 kb) [file 12882_2017_804_MOESM4_ESM.jpg]
